# Barriers and Facilitators to Genetic Service Delivery Models: Scoping Review

**DOI:** 10.2196/23523

**Published:** 2021-02-25

**Authors:** Melissa Raspa, Rebecca Moultrie, Danielle Toth, Saira Naim Haque

**Affiliations:** 1 RTI International Research Triangle Park, NC United States

**Keywords:** genetics, telehealth, genetic services, rare diseases

## Abstract

**Background:**

Advances in diagnostics testing and treatment of genetic conditions have led to increased demand for genetic services in the United States. At the same time, there is a shortage of genetic services professionals. Thus, understanding the models of service delivery currently in use can help increase access and improve outcomes for individuals identified with genetic conditions.

**Objective:**

This review aims to provide an overview of barriers and facilitators to genetic service delivery models to inform future service delivery.

**Methods:**

We conducted a scoping literature review of the evidence to more fully understand barriers and facilitators around the provision of genetic services.

**Results:**

There were a number of challenges identified, including the limited number of genetics specialists, wait time for appointments, delivery of services by nongenetics providers, reimbursement, and licensure. The ways to address these challenges include the use of health information technology such as telehealth, group genetic counseling, provider-to-provider education, partnership models, and training; expanding genetic provider types; and embedding genetic counselors in clinical settings.

**Conclusions:**

The literature review highlighted the need to expand access to genetic services. Ways to expand services include telehealth, technical assistance, and changing staffing models. In addition, using technology to improve knowledge among related professionals can help expand access.

## Introduction

Advances in diagnostic testing and treatment options for genetic conditions have led to increased demand for genetic services in the United States. The gateway to genetic services is through multiple paths. For example, newborn screening programs test all infants shortly after birth for a variety of genetic conditions. Other avenues include clinical diagnosis from a broad array of specialists, such as neurologists, oncologists, and geneticists. We sought to understand how genetic services are provided and identify the most cost-effective methods of meeting growing needs for services. Understanding the current delivery models being used can help strengthen the long-term follow-up of individuals identified with genetic conditions and, ultimately, improve outcomes for patients and families.

The goal of this study is to identify evidence from the literature regarding the challenges and potential solutions to improve service delivery models. We sought to understand the following research questions: (1) What are the current practice methods and models for genetic services? (2) What are the barriers or challenges presently being encountered that impede the provision of timely genetic services? and (3) What are the best practices, lessons learned, and service offerings that can inform future efforts?

## Methods

We conducted a series of 3 iterative literature searches with increasing specificity of search terms. The searches enabled the research team to amass a broad base of literature related to genetic service models spanning from genetic services delivered in early infancy, as part of newborn screening, to services delivered in adulthood, with the onset of health conditions such as cancer. This was intended to explore models that may have been implemented successfully in other health areas or populations that could potentially be of interest for application or adoption in the United States. Search parameters were consistent in all 3 searches (Textbox 1), and search terms were altered for each search (Textbox 2).

In total, 2 researchers reviewed all search results for initial inclusion. Researchers independently reviewed each abstract and made notations related to reported challenges and potential solutions regarding the delivery of genetic services. These notations guided inclusion and exclusion decisions. Researchers reviewed each other’s notations, discussed any areas of disagreement, and ultimately came to a consensus on whether the article should be obtained and included in the review. Full-text articles were obtained for all search results that met these initial criteria; each article was reviewed, and themes were extracted.

**Search parameters of the scoping review.**

**Language**

- English

**Period**

- 2010-2020

**Geographic population**

- United States and international

**Databases**

- PubMed 
- Embase
- PsycINFO 
- Web of Science (includes Science Citation Index Expanded, Social Sciences Citation Index, Conference Proceedings Citation Index-Science, and Conference Proceedings Citation Index-Social Science and Humanities)

**Search terms of the scoping review.**

**Search 1**

- Genetics AND service delivery OR model

**Search 2**

- Genetic(s) service(s) provision OR genetic(s) service(s) delivery OR genetic(s) service(s) delivery model(s) OR genetic health care service(s) delivery OR genetic(s) support delivery OR genetic(s) support model(s) OR genetic(s) service(s) delivery structure OR genetic(s) services delivery system OR genetic(s) delivery of health care OR genetic counseling services OR genetic diagnostic services

**Search 3**

- Pediatric genetic counseling OR newborn screening follow-up AND genetic(s) service(s) provision OR genetic(s) service(s) delivery OR genetic(s) service(s) delivery model(s) OR genetic health care service(s) delivery OR genetic(s) support delivery OR genetic(s) support model(s) OR genetic(s) service(s) delivery structure OR genetic(s) services delivery system OR genetic(s) delivery of health care OR genetic counseling services OR genetic diagnostic services

## Results

The search yielded 187 unique abstracts or references (Table 1). Of these, 112 articles were reviewed.

After an initial review, 93 articles related to genetic service models from across the 3 searches were selected for a full-text review. Three researchers categorized the articles by theme together and carried out full-text reviews. Table 2 outlines the articles included across the 3 major themes that were incorporated into the final review.

**Table 1:** Number of articles selected to be reviewed from search results.

Search	References in search results (n=187), n (%)	Mention of challenges in abstract/title (n=8), n (%)	Mention of solutions in abstract/title (n=85), n (%)	Mention of challenges and solutions in abstract/title (n=19), n (%)	Number of articles retrieved for full-text review (n=112), n (%)
Search 1: broad	90 (48)	2 (25)	44 (52)	14 (74)	60 (54)
Search 2: genetic services specified	67 (36)	3 (38)	31 (37)	5 (26)	39 (35)
Search 3: pediatric	30 (16)	3 (38)	10 (12)	0 (0)	13 (12)

**Table 2:** Number of articles by theme.

Category	Number of articles reviewed for solutions		
	Telegenetics (n=37), n (%)	Training, education, and awareness (n=34), n (%)	Infrastructure/workflow (n=36), n (%)
Search 1: broad	23 (62)	18 (53)	15 (42)
Search 2: genetic services specified	2 (5)	5 (15)	7 (19)
Search 3: pediatric	12 (32)	11 (32)	14 (39)

### Challenges and Barriers Identified in the Literature

#### Limited Number of Genetics Specialists

The shortage of genetics professionals coupled with a rapidly growing need has been described as one of the biggest challenges facing the field [1]. There is approximately 1 genetics professional per 300,000 individuals in the United States [2]. Radford et al [3] reported similar deficits in staffing, noting that 2500 certified genetic counselors practice in the United States, equating to 8.1 genetic counselors per 1 million population. Rural areas and certain states experience these shortages more profoundly [3,4]. Increased demand for genetics professionals is based on a few factors, including the growing number of conditions that can now be identified as having a genetic cause, affordability of testing technologies, and demand for genetic counselors in more diverse clinical settings [3,5].

In a survey of state newborn screening coordinators within the Southeast Regional Newborn Screening and Genetics Collaborative, close to half of the respondents indicated that the adequacy of the number of genetic counselors, dietitians, and medical or biochemical geneticists was minimal to insufficient [6]. One study related to newborn hearing loss reported that the deficit in the workforce has resulted in poor follow-up of patients. Al-Mulki and Todd [7] sought to explore staffing and loss to follow-up in newborns who did not pass hearing screening. The authors found that follow-up rates were higher when a full-time navigator position was filled [7].

However, as the need for these professionals continues to grow, the number of clinical geneticists entering the field is decreasing, with approximately 50% of medical genetics residency positions unfilled each year [1]. In some areas, genetic counseling positions have been difficult to fill [8].

#### Wait Time for and Length of Genetics Appointments

Two of the main challenges related to the delivery of genetic services were the time intensiveness of providing genetic counseling and wait times for appointments. Despite the fact that genetic service delivery models have changed over the years and fewer visits per patient are common, this is not the case for all areas of genetic counseling; genetic counseling continues to be a time- and resource-intensive process [1]. In a survey of cancer genetic counselors, 92% were spending fewer than 75 min for initial counseling, although recommendations suggest up to 3 hours [9]. Genetic counseling sessions have been reported to take longer when conducted by a genetics professional compared with another specialist [10]. Time was also mentioned in a study by Brierley et al [11], where nongenetics professionals required more time than they had available to perform genetic counseling and testing. In addition to the length of genetics appointments, several studies have discussed wait time as a significant barrier to accessing services, which is often linked to limitations in the workforce and geography [1,4,12].

#### Delivery of Services by Nongenetics Providers

Several studies have explored the delivery of genetic services by nongenetics professionals and the resulting challenges and impacts on patients [1,4,11,13-15].

Although primary care providers are well positioned to recognize whether genetic tests or referral to genetic counseling is appropriate, there are still gaps in identifying the need for genetic services [1]. In addition, nongenetics professionals do not always follow guidelines. This was indicated in one survey conducted in Florida that focused on pretest genetic counseling services for hereditary breast and ovarian cancer [15]. Another study reviewed 200 patients with breast or ovarian cancer who met the criteria for genetic testing. Lacking a referral from the attending oncologist was cited as the biggest barrier for 30% of the patients who did not receive adequate testing or counseling [16].

Patients can experience adverse outcomes from delivery of services by nongenetics providers. A web-based survey and structured telephone interview with genetic counselors revealed patients’ negative outcomes on receiving genetic services from nongenetics professionals in Minnesota. These outcomes included adverse psychosocial effects, medical mismanagement, inadequate counseling, negative shifts in attitudes toward medical providers, and unnecessary use of resources [13]. Another study invited genetic counselors from the National Society of Genetic Counselors (NSGC) Cancer Special Interest Group to submit cases of adverse outcomes of genetic counseling and testing performed by nongenetics professionals. The list included incorrect genetic tests ordered, misinterpretation of results, inadequate genetic counseling, unnecessary testing and surgery, distress, and false reassurance [11].

#### Reimbursement and Licensure

There are inherent challenges related to difficulties in implementing, sustaining, and scaling alternative delivery models [1,5]. Some of these challenges are because of logistical issues, including billing, reimbursement, required equipment and setup, and the inability to see the patient (ie, in the case of telephone counseling) [17]. Nonuniform delivery of services may be because of the varying state-level laws and license requirements for genetic counselors [2]. Others have described that alternative models have not been widely tested, and given the increasing role of technology, new approaches to training counselors will also need to be developed [6].

### Models to Address Barriers Identified in the Literature

#### Provider-to-Patient Telegenetics

The use of telehealth practices in the field of genetics, often referred to as telegenetics, has increased steadily in the past decade. Most often, this is through the use of video-based technology, although phone-based delivery of services to patients has also been used. A landscape review article by Terry et al [12] found that many clinics in the Mid-Atlantic region had started using telegenetics to deliver services to patients. Clinics that had used telegenetics were often located in academic or other medical institutions with a focus on prenatal and cancer services. However, in-person counseling is still a prevalent method of genetic counseling delivery. In a web-based survey of members of the NSGC Familial Cancer Special Interest Group, the face-to-face pretest and posttest model was reported as the most commonly used (92.2%) [18].

Those who have used telegenetics to deliver services to patients indicate that it allows providers to see more patients, reduces wait time, and improves costs [8,19-21]. In addition, patient satisfaction is high, with many studies citing benefits such as reduced travel time and increased convenience [22-28]. Most patients report being comfortable with using technology [22], although some still prefer in-person care, given possible technical problems or reduction in the quality of the interaction [24]. Those who are less in favor of telegenetics are often older patients [26].

A mini-review by Buchanan et al [23] identified models that use telephone genetic counseling at different points of care. This included pretest telephone counseling, posttest telephone counseling, and a model developed by a Dutch group, called *DNA-direct*, with a telephone pretest counseling model accompanied by mailed educational materials, followed by an in-person posttest consultation. Rayes et al [29] described the protocol for MAGENTA (MAking GENetic Testing Accessible), a national randomized controlled trial that will compare web-based genetic education and telephone genetic counseling for hereditary cancer genetic testing.

Outcomes reported through telegenetics are fairly well documented, with most focusing on the delivery of telegenetic counseling. When compared with baseline scores or in-person genetic service delivery, most patients reported reduced anxiety and high levels of knowledge [22]. Telephone counseling has also reported positive outcomes [30], although one study found better outcomes for video- versus phone-based genetic counseling [31].

#### Group Genetic Counseling

Another possible option for improving the efficiency of genetic services is the delivery of genetic counseling services through a group model. Two studies compared group counseling with individual counseling [32,33]. In both the studies, participants in each group reported high levels of satisfaction. Although participants in both the group and individual genetic counseling encounters reported significantly decreased patient anxiety, increased perceived personal control, decreased decisional conflict, and increased knowledge, those in the individual session reported a greater reduction in anxiety than those in the group session [32]. Woodson et al [34] described a group counseling model for an underserved population in Texas focused on women receiving counseling for hereditary breast and ovarian cancer [34]. Group genetic counseling was also reported to increase efficiency by decreasing the time spent per patient [23]. When asked to rate their preference for individual versus group counseling, however, most ranked individual counseling higher [35].

#### Provider-to-Provider Telegenetic Consultation

In this model, primary care providers have access to specialty providers to gain knowledge regarding care and monitoring of patients with special health care needs. Much of this work has been patterned after the Extension for Community Healthcare Outcomes model. This approach is gaining traction in the field of genetics [36].

#### Partnership Models Between Genetics and Nongenetics Providers

In addition to telegenetics, the use of a team-based or collaborative approach has been suggested as an alternative genetic service delivery model [37]. Kubendran et al [38] described the implementation of pediatric telegenetic services by a team comprising a geneticist, a pediatrician, and a genetic counselor. Patients reported high satisfaction with this team-based approach. A similar multidisciplinary approach was used in a pediatric metabolic practice for patients with inborn errors of metabolism [39]. This team consisted of metabolic geneticists, pediatric dietitians, clinical pharmacists, social workers, metabolic nurses, and genetic counselors who provide care and frequent follow-up to patients. An important feature that was highlighted as part of this model was the ability of the genetic counselor to build a long-term relationship with the patients and families because of the frequency of management visits. Other benefits of the multidisciplinary model include reducing the burden of frequent visits to the hospital for the patient and family to see each care provider separately and reducing the financial burden for frequent visits to the hospital because many patients travel from longer distances to access tertiary care.

Other studies have discussed a partnership model between genetic counselors and physicians in oncology [40]. One study in an oncology clinical setting evaluated the differences between a model of cancer genetic counselors in traditional clinics combined with a medical geneticist compared with genetic counselor–only appointments [41]. The authors tracked the amount of time spent on patient interaction and outpatient-related care over 9 months and found that genetic counselors performed similar activities without the medical geneticist present and spent significantly less time in appointments when they worked alone as opposed to working with a medical geneticist, which may be attributed to redundancy in services.

One study in Hong Kong used a new model to provide clinical genetic services [42]. An institute located within a hospital provided genetic diagnostic and counseling clinics that were supported by an in-house laboratory, including cytogenetics, molecular genetics, and diagnostic laboratories. This model allowed the genetics team to work closely with a variety of other providers in the hospital setting.

Partnership models with other health professionals, such as social workers and psychologists, also emerged as potential models for patient-centered genetic care. Telfair argues that social work education should include the basics of genomics because social workers may play a large role in genetic counseling, noting their ability to ensure that clients have access to counseling and testing [43]. Other authors explore partnerships with psychologists to improve care for patients and families receiving genetic services. For example, the feasibility of offering a narrative group session offered by a genetic counselor and a clinical psychologist to individuals who tested negative for Huntington disease was assessed, and participants provided overwhelmingly positive feedback about this approach [44]. Another study assessed the level of satisfaction among Brazilian mothers of children with Down syndrome who received care from a multidisciplinary team consisting of 3 counselors, 3 psychologists, a social worker, 3 physicians, and 2 nurses. Satisfaction with the information received and psychological support was high among the mothers [45].

#### Training and Educational Resources

With genetics and nongenetics professionals alike, training and educational resources were indicated as needs [2,6,14,15]. Genetic counselor training programs face challenges related to the need to expand knowledge and to meet increasing demands within the context of the various roles that genetic counselors play in academia, clinical service, and industry [5]. Moreover, as genetic services continue to become more integrated into primary care or other specialties, roles, data and database sharing, and preparation of nongenetics professionals will need to be explored [46]. Stoll et al [1] discuss training and educational interventions as solutions to assist primary care providers in improving knowledge and helping to recognize and triage patients for genetic testing and counseling services [1]. Others have noted that professionals can incur liability, and patients can experience negative outcomes as a result of inadequately trained providers [6].

#### Embedding Genetic Counselors Within Clinical Settings

Another approach to the delivery of genetic services is to embed genetic services or genetic counselors into nongenetic clinical practices. In 2012, Battista et al [46] carried out a literature review of genetic services in North America, Europe, and Australia, highlighting the advantages of integrating genetic services into primary care. For rare genetic conditions, the study’s authors recommended the use of multidisciplinary specialist clinics or coordinated services with primary care providers. For other common genetic conditions, such as genetics-related cancers, interprofessional collaboration between geneticists and other medical providers seemed sufficient for coordinating care.

One study assessed the attitudes and barriers of incorporating a genetic counselor into a cystic fibrosis clinic in New York [47]. Among center directors and clinic coordinators who responded to the survey, 84% indicated that genetic counselors provide a valuable service in the clinic and 85% endorse this relationship (as indicated by a *recommend* or *strongly recommend* response).

This model has been translated into and tested in multiple clinical settings, including cancer care. In a gynecologic oncology clinic in Ohio, a higher number of patients were referred for genetic consultation after changing to a genetics-embedded model; referrals increased from 21% to 44% of patients [48]. In addition, time from referral to scheduling a genetics appointment was reduced. A similar finding was reported in a specialized gastrointestinal cancer center [49]. A 223% increase in the number of patients receiving genetic services occurred after integrating genetic counselors into the gastrointestinal cancer center. Pederson et al [50] conducted a retrospective review of patterns of genetics referrals, compliance, and testing over a 2-year period before and after a genetic counselor was embedded within a breast surgery clinic [50]. Not only did the likelihood of being referred to genetic services increase by 49%, but a greater number of patients were also more likely to go through with a genetic counseling appointment and take part in a genetic counseling session before surgery. Notably, the authors reported a 31% reduction in time to treatment between the 2 periods.

#### Use of Genetic Counseling Assistants

The use of genetic counseling assistants (GCAs) has also been explored as a solution to the shortage of genetic counselors. Pirzadeh-Miller et al [51] explored the use of GCAs from a survey distributed to certified genetic counselors, GCAs, and genetic counseling program directors [51]. Genetic counselors were able to see more patients when they had a GCA (58.5% more patients with a ratio of 3 genetic counselors to 1 GCA), and all genetic counselors surveyed reported an increase in efficiency in patient care or productive time utilization. GCA responsibilities endorsed by genetic counselors included entering data, shipping test kits, performing administrative tasks, and ordering supplies. However, fewer than half of genetic counselors reported endorsing GCAs for calling patients with test results or formulating results letters for patients. Of the genetic counselor respondents, 90% reported that the GCA profession could lead to a genetic counseling career. GCA respondents reported similar duties, and most GCAs (86%) were interested in a genetic counseling career.

In a recent survey, members of the NSGC responded to a survey regarding the role of GCAs and the impact they may have on the genetic counseling profession [52]. Themes that arose for how GCAs could change their practice included increased time available for higher-level duties, higher patient volumes, and increased efficiency. There was consensus among respondents on what GCAs should be doing, but the scope and guidelines for the role of GCAs have not been clearly defined. Respondents who currently did not use GCAs had greater concerns about the role of, and burden of supervising, GCAs compared with respondents who were currently working with GCAs.

#### Use of Health Technologies and Patient Educational Tools

A handful of studies described various technologies that are being implemented to support the delivery of genetic services. Flannery [53] identified some of these technologies, including eConsult, an internet-based communication method between primary care physicians and specialists; chatbots, which use artificial intelligence (AI) to produce interactive conversations; and a web-based platform that integrates education on carrier results with personal test results. Moreover, 2 other studies endorse the use of chatbots [54,55]. Schmidlen et al [55] explored the use of chatbots for scaling up communication in genomics research, specifically for consenting to research, interacting with health care providers after receiving genomics results, and sharing genetic information with relatives.

Kearney et al [56] describe the current applications of AI in genomics and genetic counseling and the potential for continued and increased integration of AI in genetics. Medical genetic testing is one of the major applications of machine learning and deep learning in genomics. Another use of machine learning is in the development of clinical decision support tools. Examples cited include tools that could search literature based on molecular profile, image recognition to identify a genetic diagnosis based on a patient’s phenotype, or matching genetic diseases to the identified symptoms.

#### Implementation and Evaluation of Alternate Service Delivery Models

A few recent papers explored how to best implement or evaluate the use of alternate service delivery models. For example, Chou et al [57] developed a genetic services assessment tool consisting of 16 quality indicators for states to use to evaluate public health genetic services. The major domains identified include (1) structural metrics that are key components to quality genetic services: workforce, training and education, information systems, and types of programs provided; (2) clinical process metrics affecting quality genetic services: patient-provider interactions, care coordination and management, quality assurance and improvement mechanisms, and care provision and service delivery (metrics describe patient-provider interactions, continuity of care, quality programs, and performance tracking); and (3) outcome measures of quality genetic services: process-of-care outcomes (screenings and referrals rather than health outcomes). The authors suggest that this tool will help states identify key areas for improvement and quantify progress made.

Other quality improvement initiatives have underscored the potential for new and innovative frameworks or learning collaboratives to address existing shortcomings and challenges in genetic service delivery. Russ et al [58] described one such quality improvement approach that was used to reduce the significant loss to follow-up after newborn hearing screening. Before implementation, more than half of the children were lost to follow-up after a newborn hearing screening. However, after using this quality improvement approach, new strategies were adopted, and teams reported decreasing loss to follow-up rates.

Textbox 3 presents an overview of the challenges and possible solutions outlined in the literature.

**Challenges and possible solutions.**

**Limited number of genetics specialists**

- Patient-to-provider telegenetics
- Group genetic counseling
- Use of genetic counseling assistants
- Use of health technologies and patient educational tools
- Implementation and evaluation of alternate service delivery models

**Wait time for and length of genetics appointments**

- Patient-to-provider telegenetics
- Use of health technologies and patient educational tools
- Implementation and evaluation of alternate service delivery models
- Embedding genetic counselors within clinical settings

**Delivery of services by nongenetics providers**

- Provider-to-provider telegenetic consultation
- Partnership models between genetics and nongenetics providers
- Training and educational resources
- Use of health technologies and patient educational tools
- Implementation and evaluation of alternate service delivery models

**Reimbursement and licensure**

- Use of genetic counseling assistants
- Implementation and evaluation of alternate service delivery models

## Discussion

### Expand Access to Genetic Services

The literature review pointed out several challenges that are currently facing the field of genetics. Those that cause the most concern for access to genetic services are the limited number of genetics specialists available to meet the growing demand for services and the long wait times needed to get appointments. However, several possible solutions highlighted in this literature review could be implemented to address these challenges.

The use of telehealth is one of the main ways to increase access to genetic services. On the basis of the literature review, there is ample evidence to support the use of telehealth. Several challenges can be addressed through the use of telehealth. First, because of the vastness of the United States and the dearth of genetic service providers, each of the genetic services centers serves a large catchment area. Thus, there are geographic barriers to access to care. Telehealth can help alleviate these barriers. Second, many states have a large Spanish-speaking population. However, there is a shortage of Spanish-speaking providers. Through telehealth, bilingual providers can expand their reach. In addition, telehealth can facilitate the use of remote translation services. Finally, there is a nationwide shortage of genetic service providers across the continuum. Telehealth expansion can help with workforce issues by obviating the need for staff to travel to multiple locations. In addition, telehealth can help with load-balancing staff across locations.

Increasing the use of telehealth services, however, may require additional assistance or planning. Other needs related to the expansion of telehealth may emerge. For example, providers may need access to training opportunities, to purchase equipment, or to feel prepared to implement telehealth. Support for training can come through existing resources, such as those provided by Telehealth Resource Centers [59]. Readiness assessment tools can assist with preparations to implement telehealth [60,61]. In addition, there are organizational factors associated with the implementation of telehealth. Providers need to develop a telehealth delivery plan that might include an outline for which types of visits will be used for telehealth (eg, only follow-up visits), which providers can implement telehealth (eg, dietitians and genetic counselors), and how to integrate the telehealth platform with the electronic health record. In addition, sharing ideas and recommendations on change management principles associated with telehealth, such as readiness, training, and workflow, can help the state manage implementations and save resources. Finally, it will be important to monitor the status of reimbursement for telehealth services. Although reimbursement for telehealth has expanded recently, mainly because of the COVID-19 pandemic, it remains unclear if some, all, or none of the recent changes will be permanent. Changes include the removal of geographic restrictions (including allowing telehealth from home), billing and reimbursement changes (eg, new reimbursement codes available and same fee-for-service rate as in-person visits), and the addition of phone-only service provision. Thus, coordinating with Medicaid, as well as private payers, on reimbursement for telehealth services could facilitate uptake.

In addition to telehealth, there are a few possible solutions related to clinical workflow that may improve access to genetic services. The use of GCAs has been rated favorably in many of the studies included in the literature review. For high-volume clinics, another consideration to improve clinic flow could be the use of group genetic counseling for low-risk populations followed by an individual in-person or telephone session. Another possibility is the use of patient education tools to assist with the amount of time genetic counselors spend with patients individually.

### Build Expertise and Improve Knowledge About Genetic Services Among Nongenetics Professionals

Given that fewer genetics professionals are entering the field and the current workforce issues, there is a need to build genetics knowledge and expertise among nongenetics professionals. This can be accomplished by using one or more possible approaches that were revealed during the literature review. Partnership models were used to expand access to genetic services in collaboration with nongenetics professionals, sometimes through telehealth. These solutions allow patients and families to access a genetics specialist and build relationships with nongenetics providers, which enhances their expertise. This could be accomplished through outreach to primary care settings of existing patients who see a genetics professional. Similarly, provider-to-provider telegenetic consultation is a strategy that can be employed to pair genetics providers with nongenetics providers. Through consultation with experts, nongenetics professionals gain a better understanding of their patients and are better able to care for their unique needs. Both these approaches would also help to address the noted challenges of nongenetics providers delivering services without the support of a genetics specialist (eg, ordering the wrong genetic test or panel).

Another possible solution to improve the knowledge and expertise of nongenetics professionals is to embed genetics providers within primary care settings. Typically, genetic counselors are hired to be part of primary care settings to assist with referrals and provide long-term support to patients. This approach, though, may be more challenging to implement, given there is still an insufficient number of genetic counselors, although the field is growing. It may also be more difficult, given that the state does not have the authority to direct how primary care providers structure their office staff.

### Conclusions

The literature review illuminated the challenges and identified possible solutions that could be implemented to improve the delivery of genetic services. Options that include telehealth applications may be the most straightforward and immediate option for genetic centers to pilot. However, a more long-term investment will be to complement telehealth models with the education of nongenetics professionals. Given the likely continued shortage of providers in the field of genetics, a transdisciplinary approach will be needed to build the expertise of primary care providers and other health care professionals to best serve the needs of patients and families.

## Abbreviations

AI: artificial intelligence
GCA: genetic counseling assistant
NSGC: National Society of Genetic Counselors
